# Energy extraction from dark Fe^3+^ in A_2_Sc_2_B_4_O_11_:Fe^3+^, Yb^3+^ (A = Sr, Ba) toward promoted NIR luminescence and pc-LED light source for multifunctional applications

**DOI:** 10.1038/s41377-026-02284-8

**Published:** 2026-05-09

**Authors:** Dechao Yu, Haisheng Liu, Mengting Lv, Benchun Li, Yayun Zhou, Xinxin Han, Dawei Zhang

**Affiliations:** 1 Engineering Research Center of Optical Instrument and System, Ministry of Education and Shanghai Key Lab of Modern Optical System, University of Shanghai for Science and Technology, Shanghai, China; 2 Guangdong-Hong Kong-Macao Joint Laboratory for Intelligent Micro-Nano Optoelectronic Technology, School of Physics and Optoelectronic Engineering, Foshan University, Foshan, China; 3 MOE Key Laboratory of New Processing Technology for Non-ferrous Metals and Materials, Guangxi Key Laboratory of Processing for Non-ferrous Metals and Featured Materials, Guangxi University, Nanning, China

**Keywords:** Optical materials and structures, Inorganic LEDs

## Abstract

New generation of Cr^3+^-free eco-friendly phosphors (no risk of Cr^3+^ → Cr^6+^ oxidization toxicity) are highly sought to develop broadband NIR light sources. As an essential element for body health, Fe^3+^ ion would be an exceptional alternative in strong octahedral crystal field. Here, the Fe^3+^ activators were utilized in orthoborate-pyroborate A_2_Sc_2_B_4_O_11_ for creating novel NIR-emitting phosphors. A broad absorption over 240–450 nm due to O^2-^ → Fe^3+^ charge transfer transition was recorded for Sr_2_Sc_2_B_4_O_11_:Fe^3+^ (SSBO:Fe^3+^) at 370 nm and Ba_2_Sc_2_B_4_O_11_:Fe^3+^ (BSBO:Fe^3+^) at 355 nm. Resultant NIR emissions with large full width at half maximum about 170 nm were obtained for SSBO:Fe^3+^ peaked at 975 nm and BSBO:Fe^3+^ at 930 nm. The unique excitation of Fe^3+^ doping towards near-ultraviolet (near-UV) region was initially achieved for potential advantage of coupling a mainstream UV chip. Codoping of Yb^3+^ into A_2_Sc_2_B_4_O_11_:Fe^3+^ made emission peak red-shift towards 1000 nm and ~ 160-fold enhancement in the integral intensity owing to a robust energy extraction from the major dark (nonluminous) Fe^3+^. The optimized SSBO:0.02Fe^3+^,0.15Yb^3+^ exhibited considerable internal and external quantum efficiency ~ 78% and 48%, respectively. Compared to the luminescence thermal stability of ASBO:Fe^3+^ (32%@373 K, i.e., sustaining 32% of its room-temperature emission intensity at 373 K), the Yb^3+^ codoping endowed much superior stability > 63%@373 K, and additional temperature sensing with relative sensitivity ~ 1.5% K^−1^ at 423 K. Ultimately, by coating the novel phosphors onto UV ~ 365 nm chips, the home-made pc-LEDs were applied in night vision, food inspection, biomedical imaging, and spectroscopy analysis.

## Abstract (web summary)

Robust energy extraction from dark Fe^3+^ via efficient Fe^3+^→Yb^3+^ energy transfer in A_2_Sc_2_B_4_O_11_:Fe^3+^,Yb^3+^ (A = Sr, Ba) phosphors yields 160-fold enhanced NIR luminescence, enabling eco-friendly pc-LEDs for versatile non-destructive inspection.

## Graphical abstract

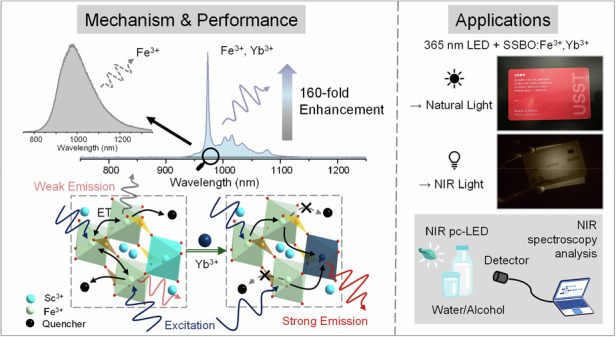

## Introduction

Nowadays near-infrared (NIR) light (approximately 700–2500 nm) is popularly used in various fields, such as night vision, non-destructive detection, food analysis, biomedicine, indoor planting, etc^1–9^. Compared to visible light, NIR light features advantages of invisibility to the unaided human eye, intense penetration ability and characteristic absorption by specific (organic) substances, which significantly endows novel NIR-emitting materials and photoelectric devices with profound interests and urgent demands. Amongst all the mass-market NIR light sources like tungsten halogen lamp, supercontinuum laser, and laser diode, NIR-emitting phosphor-converted light-emitting diodes (pc-LEDs) combine almost all the impressive advantages of LED chips and short-wave infrared phosphors, such as a compact size, tunable broad emission bands, high efficiency, and long-term services. In practice, development of novel and efficient NIR-emitting phosphors with tunable absorption/emission properties has become essential for promoting the fabrication and application of advanced NIR pc-LED light source^10–13^.

Rare-earth (RE) elements with 4 f^*n*^ electronic structure (*n* = 1–14 for RE = Ce-Lu) have been typically doped as colorful activators into a large number of host matrices to efficiently convert ultraviolet (UV)/visible photon absorption into NIR photon emission. However, due to the forbidden nature of intra-4f transitions, the RE^3+^-activated NIR luminescent materials exhibit intrinsic linear absorption/emission, greatly limiting their practical applications. Although some divalent RE ions like Eu^2+^ can feature broadband absorption/emission because of the (partially) allowed 4f-5d inter-configurational transitions, their emission wavelengths are in the vicinity of far-red light region, thus leading to poor penetration performance in biological tissues and organic substances^14–16^. At present, trivalent chromium (Cr^3+^) ion as an activator is popular for its efficient tunable broadband NIR luminescence in about 650–1200 nm as it is doped into octahedral sites with a relatively weak crystal field^17–20^. The Cr^3+^ ion has a unique 3d^3^ electronic configuration with a spin-allowed ^4^A_2g_ → ^4^T_1g_ absorption and a ^4^T_2_ → ^4^A_2_ emission. Although the Cr^3+^-doped NIR-emitting phosphors are intensively studied up to now, the Cr^3+^ ion readily undergoes oxidation to form chromium ions of higher valence states, i.e., Cr^6+^, which on one hand leads to a marked decline in NIR emission efficiency^2,10,21^, and on the other hand results in a substantial increase in toxicity^22,23^. Therefore, there still exist big challenges to develop novel NIR-emitting phosphors as well as pc-LED NIR sources to meet the emerging application requirements.

As an essential element for maintaining human body healthy, transition metal ion Fe^3+^ is also notable for its environmental friendliness and stability^24^. Although Fe^3+^ ion has traditionally been recognized as an efficient luminescence quencher due to its low-lying d-d transition states providing non-radiative relaxation pathways, a recent paradigm shift has emerged^25,26^. Rather distinct from this earlier consensus, recent studies have demonstrated that in certain novel host lattices, Fe^3+^ dopants can serve as exceptional alternatives for efficient NIR luminescence^27,28^. Analogous to the Cr^3+^ activator, the Fe^3+^-doped materials can also radiate broadband-tunable NIR emission in a strong octahedral crystal field through the ^4^T_1_ → ^6^A_1_ transition of Fe^3+^ ion. The evolution of Fe^3+^-doped phosphors has been marked by several key milestones. Initial studies focused on fundamental luminescence verification in simple oxides, such as ZnGa_2_O_4_:Fe^3+^, which confirmed that Fe³⁺ could function as a broadband NIR emitter analogous to Cr^3+^^29^. A breakthrough was subsequently achieved in the antimonate double-perovskite family. Notably, Ca_2_InSbO_6_:Fe^3+^ demonstrated a record-breaking internal quantum efficiency (IQE) of ~ 87%, fundamentally challenging the stereotype of Fe^3+^ as a quenching center^27^. This discovery triggered a surge of interest in double perovskites, leading to the development of Sr_2_ScSbO_6_:Fe^3+^, which further highlighted the potential for superior thermal stability (81%@423 K) in this material class^30^. Beyond perovskites, the exploration extended to structurally complex hosts. For instance, Sr_9_Ga(PO_4_)_7_:0.2Fe^3+^, originally a classic host for Cr^3+^, was successfully adapted for Fe^3+^^31^. This phosphate host is distinguished by its unique cation sites and the ability to accommodate high doping concentrations, demonstrating the versatility of Fe^3+^ in diverse coordination environments. However, despite these encouraging advances, the systematic development of Fe^3+^ phosphors still encounter certain challenges. On one hand, compared to the extensive research on RE^3+^ and Cr^3+^, the fundamental understanding of Fe^3+^ luminescence appears relatively less established. In particular, the detailed mechanisms governing the energy transfer (ET) pathways, especially those among Fe^3+^ ions or between Fe^3+^ and other sensitizers/activators, require further elucidation. On the other hand, it clearly unveils that achieving tunability of Fe^3+^ excitation to align with mainstream commercial UV chips is highly challenging, thereby directly restricting the applications of Fe^3+^-doped NIR phosphors and the corresponding light source devices. Moreover, a further tunability of emission peak towards much short-wave IR region is a vital and urgent demand for developing novel categories of Fe^3+^-based NIR phosphors.

In this work, a series of mixed orthoborate-pyroborate phosphors activated by Fe^3+^ ions, i.e., novel Sr_2_Sc_2_B_4_O_11_:Fe^3+^ (SSBO:Fe^3+^) and Ba_2_Sc_2_B_4_O_11_:Fe^3+^ (BSBO:Fe^3+^), were prepared by high-temperature solid state reaction method. These Fe^3+^-activated borate phosphors can efficiently absorb UV ~ 355–370 nm light and emit broadband NIR light with peak at ~ 930–975 nm with a large full width at half maximum (FWHM) about 170 nm. It is of great interest that UV ~ 360 nm excitation of Fe^3+^ for broadband NIR luminescence was first successfully achieved in these ASBO (A = Sr, Ba) host matrixes. By codoping Yb^3+^ ions, robust ET from excited Fe^3+^ to Yb^3+^ as well as effective energy extraction from the dark (nonluminous) Fe^3+^ ions occur for a greatly enhanced NIR emission at about 1000 nm. The ET and energy extraction mechanisms were systematically studied to optimize ASBO:Fe^3+^,Yb^3+^ (A = Sr, Ba) for application of pc-LED NIR light source. Proof-of-concept applications in night vision, non-destructive detection, food inspection and NIR spectroscopy analysis well demonstrate the high-performance characteristics of these ASBO:Fe^3+^-based NIR-emitting phosphors, as well as the necessity of research on new kinds of Fe^3+^-activated phosphors.

## Results and discussion

### Structural characterization

**Fig. 1:**
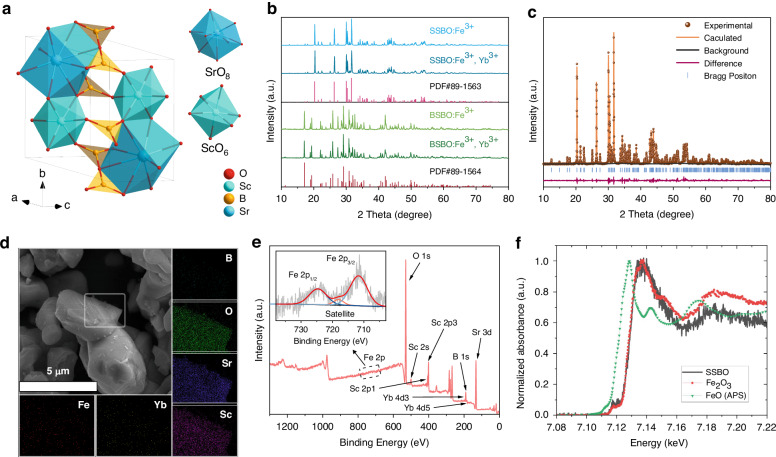
Structure characterization of ASBO:Fe^3+^ (A = Sr, Ba) phosphor. **a** Crystal structure and coordination diagram of SSBO host. **b** XRD patterns of as-prepared ASBO:0.02Fe^3+^ and ASBO:0.02Fe^3+^,0.15Yb^3+^ (A = Sr or Ba) phosphors, as well as that of SSBO (PDF#89-1563) and BSBO (PDF#89-1564) standard references. **c**–**e** Rietveld refinement **c**, SEM and elemental mapping images **d** and XPS spectra **e** of SSBO:0.02Fe^3+^,0.15Yb^3+^ representative sample. Inset of Fig. 1e specifically shows the measured Fe 2p spectrum. **f** Normalized Fe K-edge XANES spectra of SSBO:0.02Fe^3+^,0.15Yb^3+^ compared with reference standards of Fe^3+^ (Fe_2_O_3_) and Fe^2+^ (FeO, measured at APS)

Figure 1a and S1 depict the schematic diagrams of crystal structure of Sr_2_Sc_2_B_4_O_11_ (SSBO) host and that of Ba_2_Sc_2_B_4_O_11_ (BSBO) host, respectively, both belonging to mixed orthoborate-pyroborate units. The SSBO crystallizes in the triclinic space group P$\bar{1}$^32^. In the layer sandwiched by the admixture of orthoborate [BO_3_] and pyroborate [B_2_O_5_] groups, Sc atoms with six surrounding O^2-^ constitute octahedral [ScO_6_] sites and the octahedra are connected by shared edges that pass-through the borate layer, while Sr atoms occupying eight-coordinate sites [SrO_8_]. Moreover, the distance of the nearest adjacent [ScO_6_] octahedron is only 3.26 Å, which will lead to strong interaction of Fe^3+^-Fe^3+^ ion pair once Fe^3+^ doped into SSBO by replacing the Sc^3+^ sublattice sites^33^. On the other hand, the BSBO belongs to the monoclinic space group C2/c, which contains two types of Sc-centered distorted octahedra, [Sc1O_6_] and [Sc2O_6_], as pictured in Fig. S1^32^. Noted that, the contiguous [Sc2O_6_] octahedra are very close, sharing edge with each other to form a one-dimensional chain, with a closer distance of only 3.17 Å (Fig. S1a). The Ba atoms connect to eleven O atoms to form [BaO_11_] structure. Figure 1b shows the measured X-ray diffraction (XRD) patterns of ASBO:0.02Fe^3+^ and ASBO:0.02Fe^3+^,0.15Yb^3+^ (A = Sr, Ba) representative phosphors, which are all in good alignment with the diffraction peaks of standard cards, PDF#89–1563 for the SSBO host and PDF#89–1564 for the BSBO. These results indicate that there do not exist any distinguishable impurity phases, and the introduction of Fe^3+^ and Yb^3+^ ions had no significant effect on the phase structure of as-obtained products. Because the ionic radius (*r*) of Fe^3+^ with six coordinated number (CN = 6), 0.645 Å, is smaller than that of Sc^3+^ (*r* = 0.73 Å, CN = 6)^34^, the angle of Bragg diffraction peak at ~ 31.6° for plane (0–2 1) is expected to gradually shifts towards larger angles with increasing Fe^3+^ concentration from 0.001 to 0.1 for the as-obtained SSBO:*x*Fe^3+^ samples (Fig. S2a). Similarly, such diffraction angular displacement phenomenon can also be observed in BSBO:*x*Fe^3+^ (*x* = 0.01, 0.02, 0.05) in Fig. S2b, confirming the effective incorporation of Fe^3+^ into the host lattice. In addition, the measured XRD patterns of SSBO:0.02Fe^3+^,*y*Yb^3+^ (*y* = 0.01–0.3) powder samples can be well indexed into the standard card, as comparatively displayed in Fig. S2c. As shown in Fig. S2c, it is evident that with an increase in Yb³⁺ concentration, diffraction peaks, such as the one corresponding to the (0–2 1) plane at ~ 31.6°, shift towards lower angles. According to the Bragg’s law^35^, this observation validates that the larger Yb^3+^ ion (*r* = 0.868 Å, CN = 6) is likely to occupy a smaller Sc^3+^ (*r* = 0.73 Å, CN = 6) site for a favorable charge balancing in the pure phase ASBO:Fe^3+^,Yb^3+^ (A = Sr, Ba) samples. Otherwise, the substitution of Yb^3+^ ion (*r* = 0.925 Å, CN = 8) into the larger Sr^2+^ sublattice site (*r* = 1.26 Å, CN = 8) will continuously shift towards large angles, thereby leading to severe charge imbalance particularly at concentrated Yb^3+^ codoping. Additionally, the percentage difference in ionic radius, *D*_*r*_, can determine the sites occupied by the dopant ions, and can be expressed by the following formula^36,37^

1 $${D}_{r}=\,\frac{{R}_{s}\left({CN}\right)-{R}_{d}\left({CN}\right)}{{R}_{s}\left({CN}\right)}\times 100 \%$$

where CN represent the coordination number, and *R*_*s*_ and *R*_*d*_ represent the radii of the central and doped ions, respectively. The *D*_*r*_ between the Fe^3+^ and Sc^3+^ is calculated to be about 11.6% (CN = 6), while that between the Fe^3+^ and Sr^2+^ or Ba^2+^ is about 38.1% or 45.1% (CN = 8). Similarly, the *D*_*r*_ between Yb^3+^ and Sc^3+^ is ~ 18.9% (CN = 6), while that between Yb^3+^ and Sr^2+^ is much larger to be ~ 26.6% (CN = 8). Because there do not exist effective ionic radii of Fe^3+^ and Yb^3+^ in the eleven-coordinated environment, the *D*_*r*_ between the Fe^3+^ (Yb^3+^) and Ba^2+^ cannot be directly evaluated for comparison. In theory, the larger *D*_*r*_ will make the substitution much more difficult, and particularly a threshold value (close to) exceeding 30% would make impossible substitution. Therefore, the Fe^3+^ and Yb³⁺ dopants preferentially occupy the Sc^3+^ sites in the ASBO (A = Sr, Ba) host lattice.

To further probe the phase purity of as-obtained ASBO:Fe^3+^,Yb^3+^ (A = Sr, Ba) phosphors, XRD Rietveld refinements were carried out for the ASBO:0.02Fe^3+^ and ASBO:0.02Fe^3+^,0.15Yb^3+^ samples on basis of their precisely measured XRD patterns, as respectively shown in Fig. 1c and S3. The reliable *R*-factors (*R*_*wp*_, *R*_*p*_) and the refined crystallographic parameters were all detailed in Table S1. Just as expected, the calculated lattice parameters (*a*, *b*, *c*) and cell volume (*V*) slightly decrease as smaller Fe^3+^ ions replaced the Sc^3+^ sites (*V* ~ 220.26 Å^3^ versus 220.4 Å^3^ for the SSBO case; *V* ~ 937.26 Å^3^ versus 937.8 Å^3^ for the BSBO case), while increase significantly as larger Yb³⁺ ions substitute the Sc³⁺ sites (*V* ~ 221.354 Å³ versus 220.260 Å³ for the SSBO:Fe³⁺ case; *V* ~ 938.43 Å³ versus 937.26 Å³ for the BSBO:Fe³⁺ case). These results additionally evidence that all the prepared ASBO:Fe^3+^,Yb^3+^ (A = Sr, Ba) phosphors were well crystallized into pure phase. Figure 1d and S4 exhibit the scanning electron microscopy (SEM) and elemental mapping images of ASBO:0.02Fe^3+^,0.15Yb^3+^ (A = Sr and Ba) representatives. It can be seen that all the obtained polycrystalline phosphors were formed into irregular microscale blocks with severe agglomerations, and the constituent elements (i.e., Sr/Ba, Sc, B, O, Fe, Yb) are homogenously distributed on the surface of scanned particles. Noted that, all the imaged particles feature smooth surfaces, likely unveiling a high crystallinity achieved for the high-temperature sintered ASBO:Fe^3+^,Yb^3+^ phosphors. Moreover, Fig. 1e shows the X-ray photoelectron spectroscopy (XPS) spectra of representative SSBO:0.02Fe^3+^,0.15Yb^3+^ sample. It was found that there were two peaks at the binding energy of 183.9 eV and 191.9 eV, corresponding to the 4d_5/2_ and 4d_3/2_ components of Yb^3+^ ion, respectively^38^. Most importantly, the inset of Fig. 1e shows the characteristic peaks of Fe 2p_3/2_ and 2p_1/2_ at 711.7 eV and 724.6 eV, respectively, along with an observable satellite peak at ~719 eV. These results are in perfect agreement with the characteristics of Fe^3+^ ions^39^. To further determine the valence state of the iron ions, we conducted Fe K-edge X-ray Absorption Near-Edge Structure (XANES) measurements^40,41^. As shown in Fig. 1f, the near-edge spectrum of the sample closely resembles that of the Fe_2_O_3_ reference. Specifically, the black line (main absorption edge) of the sample is nearly identical in position (7.129 keV) and much similar in shape to that of Fe_2_O_3_ (red line), and is distinctly higher in energy than that of FeO reference (7.122 keV, green line). This confirms the absolutely dominant Fe^3+^ oxidation state in the well-synthesized samples.

### Luminescence properties

#### Luminescence properties of Fe^3+^-doped ASBO (A = Sr, Ba) phosphors

**Fig. 2:**
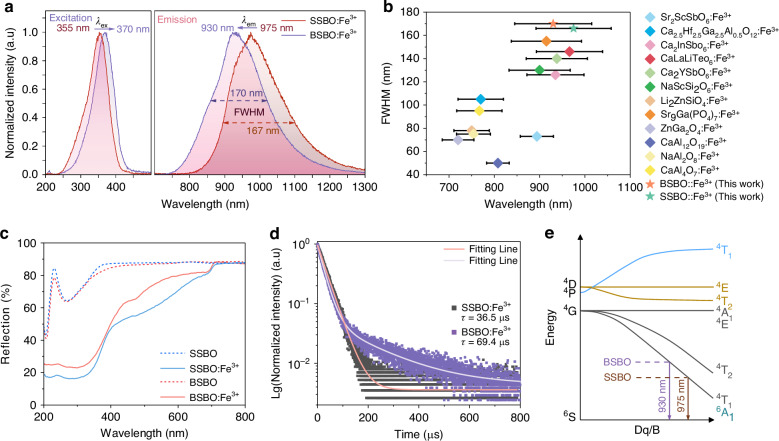
Luminescence properties of ASBO:Fe^3+^ (A = Sr, Ba) phosphors. **a** Normalized PL and PLE spectra of ASBO:Fe^3+^ (A = Sr or Ba) phosphors, respectively. **b** Comparative summary of recently reported peak wavelengths and FWHMs of Fe^3+^-activated broadband NIR-emitting phosphors. **c** Diffuse reflectance spectra compared to ASBO (A = Sr or Ba) host and **d** luminescence decay curves of ASBO:0.02Fe^3+^ (A = Sr or Ba) samples. **e** Tanabe–Sugano energy level diagram of Fe^3+^ (3d electron configuration) in SSBO and BSBO hosts

Figure 2a shows the normalized photoluminescence (PL) and photoluminescence excitation (PLE) spectra of the ASBO:0.02Fe³⁺ (A = Sr, Ba) phosphors. It can be seen that the BSBO:Fe^3+^ sample has broadband NIR emission peaked at 930 nm with a large FWHM value about 170 nm, while the SSBO:Fe^3+^ features broad NIR PL band around 975 nm with a FWHM value ~ 167 nm (right panel in Fig. 2a). These PL bands can be readily assigned to the ^4^T_1_ (^4^G) → ^6^A_1_ (^6^S) transition of Fe^3+^, which are benefited from the strong interactions with host lattice due to electron-phonon coupling effects^30^. The obvious blue-shift of BSBO:Fe^3+^ emission relative to SSBO:Fe^3+^ emission reveals that the crystal field strengths surrounding Fe^3+^ activators in BSBO host are lower than that in SSBO host^42^. When monitoring the PL peaks (λ_em_) at 930 and 975 nm for BSBO:Fe^3+^ and SSBO:Fe^3+^, broadband excitation spectra spanning over 240–450 nm can be efficiently recorded with peak wavelengths at 370 and 355 nm (left panel in Fig. 2a), respectively. These PLE bands typically originates from the O^2−^ → Fe^3+^ charge transfer (CT) transition in diverse Fe^3+^-doped hosts^27,36^. Furthermore, under optimal excitation of 355 and 370 nm (Fig. S5a), PL intensity of SSBO:0.02Fe^3+^ is much stronger than that of BSBO:0.02Fe^3+^. With increasing Fe^3+^ concentration, all SSBO:*x*Fe^3+^ samples kept consistent PL spectra in shape and peak position (Fig. S5b), had a slightly variable FWHM in 164–168 nm and reached a strongest PL intensity at *x* ~ 0.02 (Fig. S5c). These phenomena directly validate the reality of single [ScO_6_] site in the SSBO host for Fe^3+^ dopant. On the other hand, the BSBO:*x*Fe^3+^ (*x* = 0.01, 0.02, 0.05) samples also display consistent PLE bands around 370 nm, as well as broadband NIR PL bands with a slightly tunable FWHM value in about 168–174 nm (Fig. S6). In general, the BSBO:0.02Fe^3+^ sample features strongest NIR emission intensity. On basis of more spectroscopic properties of BSBO:Fe^3+^, the occupation of Fe^3+^ ions into the [Sc1O_6_] and [Sc2O_6_] octahedra could be rationally proposed in following section. Notably, as depicted in Fig. 2b, the new phosphors ASBO:Fe^3+^ have much longer PL wavelengths partially covering a desirable NIR-II window (i.e., second biological transparency window starting from 1000 nm) and larger FWHMs compared to most of recently reported Fe^3+^ NIR-emitting phosphors. That is, the mixed orthoborate-pyroborate ASBO may possess significant potential to emerge as a novel series of Fe^3+^-activated broadband NIR phosphors (see comparing details in Table S2).

Figure 2c shows the diffuse reflectance spectra of ASBO:Fe^3+^ (A = Sr, Ba) phosphors as compared with the hosts, where, well corresponding to the PLE spectra (Fig. 2a), the strong absorption bands in 250–450 nm can be identified due to the O^2−^ → Fe^3+^ CT transition. On basis of the diffuse reflectance spectral data, optical band gap (*E*_*g*_) of SSBO:Fe^3+^ and BSBO:Fe^3+^ phosphors can be evaluated using the Tauc and Kubelka-Munk equations (Eqs. S1 and S2), as shown in Fig. S7. According to the previous report, the host material is characterized as an indirect bandgap semiconductor, which is consistent with our experimental observations^43^. The large *E*_*g*_ of SSBO ~ 3.3 eV and that of BSBO ~ 3.2 eV would provide reliable support for the energy level regulation of Fe^3+^. Notably, the typical d-d transitions from the ^6^A_1_ ground state to the ^4^T_2_ (^4^D) and ^4^T_2_, ^4^T_1_(^4^G) metastable states of Fe^3+^ should appear within the range of 400–600 nm and 600–1100 nm, respectively, albeit hardly discernable (Fig. 2c) just owing to their spin and parity forbidden properties. Experimentally, the d-d transition of Fe^3+^ will be more observable for the sample with diluted Fe^3+^ dopants^27^, as depicted by PLE spectra of SSBO:*x*Fe^3+^ (*x* = 0.001, 0.005, 0.01; λ_em_ ~ 975 nm; Fig. S5d). It is of great interest that the UV excitation peaks of ASBO:Fe^3+^ (A = Sr, Ba) are located in about 355–370 nm, which are superior to most of reported Fe^3+^ NIR-emitting phosphors (CT excitation shorter than 350 nm; Table S2), as ultimately combined with the mainstream (high-power) UV LED chips for application of pc-LED NIR light source. Therefore, it can be speculated that, besides the possible tunability of ^4^T_1_ (^4^G) → ^6^A_1_ (^6^S) emission of Fe^3+^, regulation of O^2−^ → Fe^3+^ CT absorption would be feasible by gradually varying the (Sr/Ba)_2_Sc_2_B_4_O_11_ solid solutions, even towards near-UV/blue region by employing some other alkaline-earth metal ions (a future work in our group).

Figure 2d exhibits decay curves of ASBO:0.02Fe^3+^ (A = Sr or Ba) phosphors monitoring their NIR emission peak under optimal excitation, respectively. The curve of SSBO:0.02Fe^3+^ can be well fitted using a mono-exponential function (Eq. 2), while that of BSBO:0.02Fe^3+^ only can be fitted by a bi-exponential function (Eq. 3) as following^3,20^

2 $$I=A\exp \left(-\frac{t}{\tau }\right)$$

3 $$I={A}_{1}\exp \left(-\frac{t}{{\tau }_{1}}\right)+{A}_{2}\exp \left(-\frac{t}{{\tau }_{2}}\right)$$

where *I* is the PL intensity, and *A*, *A*_1_ and *A*_2_ are constants. *τ* is the lifetime calculated by Eq. 2, and ${\tau }_{1}$ and ${\tau }_{2}$ are the rapid and slow lifetimes of exponential components by Eq. 3, respectively. As a result, the peak emission of SSBO:0.02Fe^3+^ had a much short (average) lifetime ~ 36.5 *μ*s in comparison with that of BSBO:0.02Fe^3+^ sample (69.4 *μ*s, Fig. 2d). The mono- and bi-exponential decay behaviors may result from the fact that there is only one type of octahedron [ScO_6_] site for Fe^3+^ dopant in SSBO (Fig. 1a), but two [ScO_6_] sites in BSBO (Fig. S1b). Moreover, the decay curves of SSBO:0.02Fe^3+^ and BSBO:0.02Fe^3+^ monitoring NIR emission at different wavelengths (Fig. S8) can evidence the above speculations: over the whole PL spectra, the SSBO:0.02Fe^3+^ sample does feature mono-exponential decay behaviors with almost equal lifetimes about 36.0 *μ*s (Fig. S8a), while the BSBO:0.02Fe^3+^ behaves obvious bi-exponential decay with variable lifetime from 107.3 to 28.5 *μ*s by varying PL wavelength from 850 to 1100 nm (Fig. S8b; see detailed fitting parameters in Table S3). These results indicate that there do exist multiple occupation sites for Fe^3+^ activators in the BSBO host, such as the two replaceable [ScO_6_] sites. The PL spectrum of BSBO:0.02Fe^3+^ was well fitted by a sum of two Gauss functions (Fig. S9a), where two broad peaks are derived with maxima at 10411 cm^−1^ ( ~ 961 nm) and 10699 cm^−1^ ( ~ 935 nm), likely stemming from substitution of Fe^3+^ into the two different [ScO_6_] sites. Meanwhile, monitoring the different PL wavelengths, the recorded PLE spectra of BSBO:0.02Fe^3+^ (Fig. S9b) reveal that the excitation peak is located at 368 nm at λ_em_ ~ 850 nm, and then shifts to longer wavelength ~ 375 nm at longer λ_em_ around 1100 nm, clearly indicating that the diverse occupation of Fe^3+^ active sites will endow distinctive luminescent properties for broadband NIR emission and light sources. Low-temperature (80 K) PL spectra (Fig. S10) were employed to discern the emission centers. Constant peak positions and reduced bandwidths are observed for SSBO: Fe^3+^ upon cooling, characteristic of homogeneous single-center emission. In contrast, a distinct spectral shift occurs in BSBO: Fe^3+^, suggesting a temperature-induced redistribution of excited states between overlapping Fe^3+^ sites. Gaussian deconvolution of the 80 K emission spectrum for BSBO: Fe^3+^ yields two sub-peaks at 10646 cm^−1^ ( ~ 939 nm) and 10412 cm^−1^ ( ~ 960 nm), consistent with the room-temperature results (Fig. S10c). Notably, it seems that the low-energy component (~ 10412 cm^−1^) experiences more severe thermal quenching at room temperature, leading to an intensity reversal between 80 K and 298 K. This temperature-dependent spectral evolution, combined with the consistent dual-peak fitting results, provides robust evidence confirming the multi-site nature of BSBO: Fe^3+^. However, the specific assignment of these peaks to the crystallographic Sc1/Sc2 sites requires a quantitative analysis of the crystal field strength, which will be discussed in conjunction with the energy level diagram below.

Herein, it is worthy of noticing that the lifetimes of ASBO:0.02Fe^3+^ samples are all much shorter than the reported millisecond lifetime of Fe^3+^-activated phosphors^30,36^. These results suggest that most of excited Fe^3+^ might be severely quenched in the synthesized ASBO:Fe^3+^ polycrystalline phosphors, not just because of the concentration quenching (CQ) effects. That is, an in-depth understanding of PL mechanisms as well as to improve the ASBO:Fe^3+^-based NIR-emitting phosphors are critical for the desirable pc-LED applications. Generally, for the sample with concentrated Fe^3+^ ions, the CQ effects will happen as the excited energy of Fe^3+^ will be fast transferred to impurities/defect centers for efficient non-radiative relaxation. To elucidate the CQ effects and to analyze the ET mechanisms of Fe^3+^, critical distance (*R*_*c*_) was estimated by^44^

4 $${R}_{c}\,\approx 2{\left(\frac{3V}{4\pi {x}_{c}N}\right)}^{\frac{1}{3}}$$

where *x*_*c*_ is the critical concentration, *N* denotes the number of sites within a unit cell, and *V* represents the unit cell volume. Here *x*_c_ = 0.02, then *R*_c_ is obtained to be 27.61 Å (>> 5 Å), suggesting that the electric multipolar interaction rather than the exchange interaction accounts for the ET mechanisms between Fe^3+^ ions. Hence, the CQ of Fe^3+^ results from a multipolar interaction. Tanabe–Sugano diagram (3d^5^ electron configuration) in Fig. 2e depicted the energy level splitting of Fe^3+^ in an octahedral coordination environment and its radiative transition mechanisms for the ASBO:Fe^3+^ samples^42^. The ^6^A_1_ (^6^S) is ground state, the free electron level ^4^G can be split into ^4^T_1_, ^4^T_2_ and ^4^A_1_/^4^E energy states, and the ^4^D split into the ^4^E and ^4^T_2_ states. The energy states of ^6^A_1_, ^4^A_1_/^4^E, and ^4^E (^4^D) are not affected by crystal field strength. Although the ^4^T_1_ (^4^G) → ^6^A_1_ (^6^S) transition is spin and parity forbidden, it can be partially lifted through symmetry breaking induced by low site symmetry crystal field, thereby enabling efficient broadband NIR emission^28,45–47^. It also can be found from Fig. 2e that the stronger crystal field (Dq/B) will result in the larger peak wavelength of Fe^3+^ NIR emission band. The relationship between the crystal field strength (*Dq*) and the bond length (*R*) between the central ion and the ligand ion can be described as following formula^19^

5 $${Dq}=\,\frac{Z{e}^{2}{r}^{4}}{6{R}^{5}}$$

where *Z* is the valence of the anion, *e* is the charge of an electron, and *r* is the wavefunction radius of the d orbital. Comparing the two hosts, the average Sc-O bond length (*R*) in SSBO (2.091 Å) is shorter than that in BSBO (2.102 Å). According to the Eq. 5, the longer bond length in BSBO induces a weaker crystal field strength compared to SSBO. For Fe^3+^ ions (d^5^ electron configuration), a weaker crystal field increases the energy of the excited state (^4^T_1_), thereby enlarging the energy gap (^4^T_1_ to ^6^A_1_). Consequently, a blue-shift is observed in the emission spectrum of BSBO:Fe^3+^ compared to SSBO:Fe^3+^, which aligns well with the experimental results. Furthermore, based on this equation, the specific site occupation in BSBO can be rationally assigned. The average Sc1-O bond length (2.099 Å) is shorter than that of Sc2-O (2.104 Å). According to Eq. 5, the shorter bond length of the Sc1 site induces a stronger crystal field, which corresponds to the lower-energy sub-peak (peaking at ~960 nm or 10412 cm^−1^, as resolved in Fig. S10c). Conversely, the Sc2 site with a longer bond length is assigned to the higher-energy sub-peak (~939 nm or 10646 cm^−1^).

**Fig. 3:**
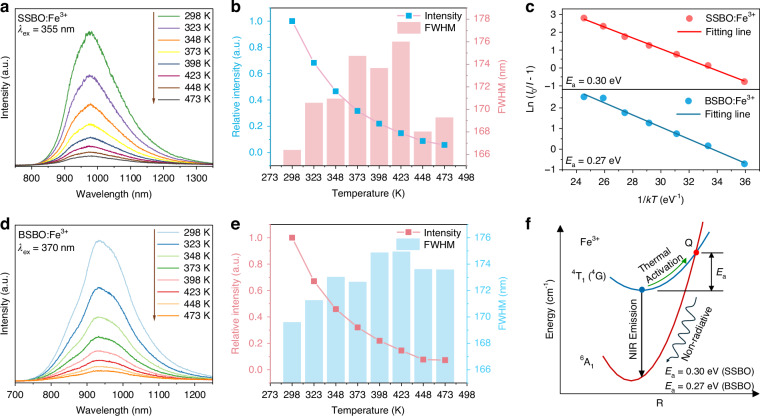
Luminescence thermal stability of ASBO:Fe^3+^ (A = Sr, Ba) phosphors. Temperature-dependent emission spectra of **a** SSBO:0.02Fe^3+^ and **d** BSBO:0.02Fe^3+^, and the corresponding integral PL intensity (dotted line) and FWHM vales (histogram) of **b** SSBO:0.02Fe^3+^ and **e** BSBO:0.02Fe^3+^, respectively. **c** Fitting curves of ln (*I*_0_/*I*-1) and 1/*kT* coordinate relationship of SSBO:0.02Fe^3+^ and that of BSBO:0.02Fe^3+^. **f** Configurational coordinate diagram of the thermal quenching for Fe^3+^ in the ASBO (A = Sr, Ba) hosts

It is known that luminescence thermal stabilities of NIR phosphors are crucial for an ultimate application of (high-power) pc-LED. Temperature-dependent emission spectra of SSBO:0.02Fe^3+^ and BSBO:0.02Fe^3+^ in Fig. 3a, d indicate that with rising temperature the Fe^3+^ NIR emissions decrease sharply due to thermal quenching. At 373 K, the integral PL intensities of SSBO:0.02Fe^3+^ and BSBO:0.02Fe^3+^ retain about 32.0% (Fig. 3b) and 31.9% (Fig. 3e) of their initial values at 298 K, respectively. Meanwhile, as the temperature increases, the emission peaks do not shift, but the spectrum becomes markedly broadened with an enhancing FWHM value until 423 K to about 176 nm for SSBO:0.02Fe^3+^ (Fig. 3b) and to 175 nm for BSBO:0.02Fe^3+^ (Fig. 3e), respectively. These phenomena are attributed to the lattice expansion induced by rising temperature, and the resultant diminution of crystal field strength, and intensification of electron-phonon coupling effect^48^. The Arrhenius equation can be used to calculate the activation energy (*E*_a_) of thermal quenching (Fig. 3c), which helps to further understand the performances of luminescence thermal stability^49^

6 $$I=\,\frac{{I}_{0}}{1+Aexp\left(-\frac{{E}_{a}}{{kT}}\right)}$$

where *I*_0_ is the initial PL intensity recorded under ideal case where thermal quenching can be ignored, here using *I*_0_ = *I*_*T*=298 K_, *I* is the PL intensity at specific temperature *T*, *A* is a constant, and *k* is the Boltzmann constant. The *E*_a_ also refers to the energy gap between the lowest position of excited energy state and the intersection point of the excited energy state with the ground state (Fig. 3f). A large *E*_a_ means that electrons in excited state need to cross a higher barrier to relax to the ground state, having better resistance to thermal quenching. On basis of the temperature-determined integral PL intensities, the *E*_a_ value was rationally calculated to be 0.30 eV for the SSBO:0.02Fe^3+^ representative and to be 0.27 eV for BSBO:0.02Fe^3+^. Compared to the reported *E*_*a*_ values in some other Fe^3+^-activated luminescent materials (Table S2), our obtained *E*_*a*_ values are medium around 0.3 eV. However, the studied Fe^3+^ NIR-emitting phosphors in this work feature considerably large Stokes shifts in the weak structural rigidity of ASBO hosts, typically resulting in poor thermal stability^14,50^.

#### Promoted luminescence characteristics of the Fe^3+^/Yb^3+^ codoped phosphors

**Fig. 4:**
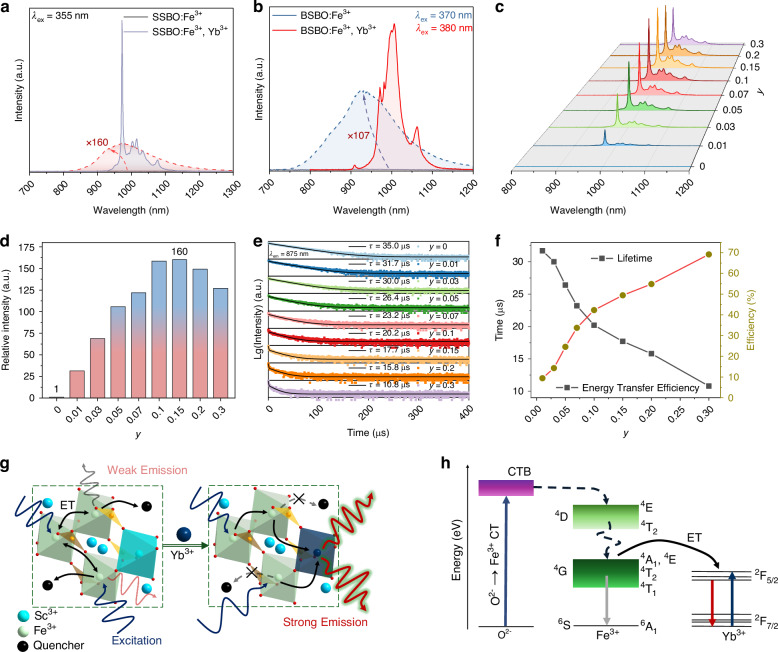
Spectral optimization of ASBO:Fe^3+^ by Yb^3+^ codoping through energy extraction. PL spectra of SSBO:0.02Fe^3+^ versus SSBO:0.02Fe^3+^,0.15Yb^3+^ **a** and that of BSBO:0.02Fe^3+^ versus BSBO:0.02Fe^3+^,0.15Yb^3+^ **b**. The PLE spectra used to determine these excitation wavelengths of BSBO:0.02Fe^3+^ and BSBO:0.02Fe^3+^,0.15Yb^3+^ are provided in Fig. S11. **c** Yb^3+^ concentration-dependent PL spectra of SSBO:0.02Fe^3+^,*y*Yb^3+^ (*y* = 0–0.3) upon 355 nm excitation, and **d** the corresponding integral intensities. **e** Decay curves of SSBO:0.02Fe^3+^,*y*Yb^3+^ monitoring Fe^3+^ emission at 875 nm as well as **f** the corresponding lifetime and ET efficiency of Fe^3+^ → Yb^3+^. **g** Scheme of energy quenching before and after the introduction of Yb^3+^ into the Fe^3+^ singly doped system. Herein, the SSBO:Fe^3+^,Yb^3+^ was used as a representative. **h** Simplified energy-level diagram of the involved CTB → Fe^3+^ → Yb^3+^ ET processes in the ASBO:Fe^3+^,Yb^3+^ (A = Sr, Ba) phosphors

Although the ASBO:Fe^3+^ (A = Sr, Ba) phosphors feature passable NIR PL performances, their emission bands well resonate with absorption of Yb^3+^ ion that has unique two multiplet manifolds for emission around 1000 nm with IQE close to 100%^49,50^. Moreover, the Yb^3+^ activator is optical inert to long wave UV/visible light. It can be speculated that codoping of Yb^3+^ into ASBO:Fe^3+^ would, at the very least, significantly challenge the above-stated Fe^3+^ NIR emission properties and the conventional excitation type of Yb^3+^ via Yb^3+^-O^2-^ CT absorption. Figure 4a, b compared the PL spectrum of ASBO:0.02Fe^3+^,0.15Yb^3+^ with that of ASBO:0.02Fe^3+^ (A = Sr, Ba). Under UV excitation, almost only the characteristic emission of Yb^3+^ was observed from Yb^3+ 2^F_5/2_ → ^2^F_7/2_ transition in 900-1200 nm with peak at 973 and 1006 nm for SSBO:0.02Fe^3+^,0.15Yb^3+^ and BSBO:0.02Fe^3+^,0.15Yb^3+^, respectively. Dashed lines in Fig. 4a, b show the integrally normalized PL spectrum of SSBO:0.02Fe^3+^ to SSBO:0.02Fe^3+^,0.15Yb^3+^ and that of BSBO:0.02Fe^3+^ to BSBO:0.02Fe^3+^,0.15Yb^3+^, respectively. Noted that, the Yb^3+^-codoping can greatly increase the overall NIR PL intensities by 160 and 107 times for SSBO:0.02Fe^3+^ and BSBO:0.02Fe^3+^, respectively. Spectral comparison in Fig. S12a indicates that the UV excitation of SSBO:0.15Yb^3+^ is peaked at 335 nm due to the Yb^3+^-O^2-^ CT absorption^51,52^, quite weak relative to that of SSBO:0.02Fe^3+^,0.15Yb^3+^ remaining at 355 nm. Under excitation of UV ~ 355 nm, the SSBO:0.02Fe^3+^,0.15Yb^3+^ sample yielded exceptionally stronger NIR emission than the SSBO:0.15Yb^3+^ sample (Fig. S12b). To further consolidate this conclusion, a similar comparative study was conducted on the BSBO system (Fig. S12c). The Yb^3+^ singly doped BSBO sample exhibits a PLE band around 254 nm, but negligible excitation response in the 300–500 nm range, just corresponding to the O^2-^ → Fe^3+^ CT transitions. Consequently, under 380 nm excitation, the BSBO:0.15Yb^3+^ sample shows virtually no luminescence, whereas the BSBO:0.02Fe^3+^,0.15Yb^3+^ exhibits intense NIR emission, interestingly having a different band shape in comparison with the PL band of BSBO:0.15Yb^3+^ excited at 254 nm (Fig. S12c). These consistent results across both host lattices just prove that the strong NIR luminescence of the Fe^3+^/Yb^3+^ co-doped sample originates exclusively from the efficient Fe^3+^ → Yb^3+^ ET processes.

However, such a tremendous enhancement in the PL intensity is inconceivable if only the original Fe^3+^-emitter completely transferred its energy to a nearby Yb^3+^ activator. Obviously, other efficient energy extraction paths from all the Fe^3+^ UV absorber were constructed among the mixing Fe^3+^ and Yb^3+^ dopants in the ASBO (A = Sr, Ba) hosts. Most interestingly, codoping of Yb^3+^ into BSBO:Fe^3+^ can also make the excitation peak red-shift to 380 nm (Fig. S11) likely due to the substitution of large Yb^3+^ into small Sc^3+^ sites, which increases Fe-O bond length and weakens electronic interaction^53,54^. This tunability will significantly promote their final applications as combined with mainstream commercial (high-power) UV LED chips. Nevertheless, the PL intensities of BSBO:Fe^3+^,Yb^3+^ samples are remarkably inferior to that of SSBO:Fe^3+^,Yb^3+^ samples, as pictured in Fig. S13. Hence, the batch of SSBO:Fe^3+^,Yb^3+^ phosphor is the key research object for pc-LED applications.

The Yb^3+^ concentration-dependent emission spectra in Fig. 4c reveal that with gradually rising Yb^3+^ concentration to 0.15 the NIR PL intensity remarkedly increases to a maximum of about 160 times in comparison with the Fe^3+^ singly doped (Fig. 4d), and then quickly decreases due to severe CQ effects at more concentrated Yb^3+^. However, the Yb^3+^ doping has little effect on the excitation performance of SSBO:Fe^3+^-based NIR-emitting phosphors (Fig. S14). Decay curves of SSBO:0.02Fe^3+^,*y*Yb^3+^ monitoring Yb^3+^ emission at 973 nm (Fig. S15) unravel that, as Yb^3+^ content increases to about 0.1, the lifetime of Yb^3+^ emission slowly decreases from 2 to 1.90 ms, while once it exceeds 0.15, the decrement of Yb^3+^ lifetime becomes marked to 1.42 ms at *y* = 0.3. This observation is highly consistent to the emission spectral quenching versus Yb^3+^ concentration (Fig. 4c). To better elucidate the ET mechanism, luminescence decay curves of SSBO:0.02Fe^3+^,*y*Yb^3+^ (*y* = 0-0.3) were carefully recorded by monitoring Fe^3+^ emission at 875 nm (non-overlapping with Yb^3+^ emission). As depicted in Fig. 4e, with increasing Yb^3+^ concentration to 0.3, the decay becomes faster with obviously changes from a mono-exponential behavior to bi-exponential features because of an accelerated ET process of Fe^3+^ → Yb^3+^. The (average) lifetime of SSBO:0.02Fe^3+^,*y*Yb^3+^ phosphors can be fitted and calculated by Eq. 2 and Eq. 3. With increasing Yb^3+^ concentration, the lifetime of Fe^3+^ emission fast decreases from 35.0 to 10.8 *μ*s, further verifying the efficient occurrence of ET process. The ET efficiency (${{\eta }}_{{\rm{ET}}}$) can be evaluated as^51^

7 $${{\eta }}_{{\rm{ET}}}=1-\left(\frac{\tau }{{\tau }_{0}}\right)$$

where *τ* and ${\tau }_{0}$ represent the lifetime of Fe^3+^ with and without Yb^3+^ codopants, respectively. The calculated *τ* and ${{\eta }}_{{\rm{ET}}}$ for the SSBO:0.02Fe^3+^,*y*Yb^3+^ (*y* = 0-0.3) phosphors are summarized in Fig. 4f. At the optimal Yb^3+^ content (*y* = 0.15), the ${{\eta }}_{{\rm{ET}}}$ of Fe^3+^ → Yb^3+^ is about 50%, which is considerable, but still cannot account for the enormous enhancement of integral PL intensities of Yb^3+^ donor ions.

To comprehensively understand the involved ET, energy extraction and PL mechanisms in the Yb^3+^ codoped ASBO:Fe^3+^ (A = Sr, Ba) phosphors, diagram of crystal structure of SSBO:Fe^3+^,Yb^3+^ representative as well as simplified energy-levels were schematically illustrated in Fig. 4g, h. For the Fe^3+^ singly doped SSBO, different Fe^3+^ ions occupying the [ScO_6_] octahedral sites have the closest distance about 3.26 Å (Fig. S1a). Especially at highly doped Fe^3+^, energy of UV-excited Fe^3+^ dopants will be quickly transferred/migrated to neighboring Fe^3+^ ions, and finally to certain quenching centers like impurities and defects that inevitably exist in the host materials or severely quenched by the electron-phonon coupling-induced non-radiative interactions (Fig. 4g). All the above processes made many dark Fe^3+^ ions and endowed the ASBO:Fe^3+^ samples with inefficient NIR radiative transitions. While for the case of Yb^3+^ codoped SSBO:Fe^3+^, the Yb^3+^ ions competitively occupy the [ScO_6_] octahedral sites with Fe^3+^ ions, and especially the concentrated Yb^3+^ will physically separate the adjacent Fe^3+^ couple ions (Fig. 4g). This separation will efficiently intercept the fast ET and energy migration between Fe^3+^-Fe^3+^ couple ions, thereby greatly extract excitation energy from the prevailing dark Fe^3+^ ions through the resonant absorption of Yb^3+^ activators (see schemes in Fig. 4h). Also benefiting from the simple two multiplets of Yb^3+^ and the resultant high emission efficiency (~ 100%), the excited Yb^3+^ activators ultimately yield massively enhanced NIR luminescence.

As schematically illustrated in Fig. 4h, in the ASBO:Fe^3+^,Yb^3+^ phosphors the CT transitions of O^2-^ → Fe^3+^ efficiently absorb UV lights, and then the excitation energy is quickly transferred to the ^4^D excited states of Fe^3+^. Following fast non-radiative relaxation, the excited electrons in the ^4^D level are decayed to the lower-lying ^4^G energy state. For the Fe^3+^-doped ASBO, energy will be continuously relaxed to the lowest ^4^T_1_ split-level, which only partially go through radiative transitions to the ^6^A_1_ (^6^S) ground state by yielding weak NIR emission around 930–970 nm (Fig. 2a). While for the Yb^3+^ codoped ASBO:Fe^3+^, energy in the ^4^G energy state of all excited Fe^3+^ ions will be resonantly transferred/extracted (from the predominant dark Fe^3+^) to the nearby Yb^3+^ ions, thereby emitting robust NIR ~ 1000 nm photons by typical transition of Yb^3+ 2^F_5/2_ → ^2^F_7/2_. Furthermore, the IQE and external quantum efficiency (EQE) of optimal SSBO:0.02Fe^3+^,0.15Yb^3+^ phosphor were measured to be about 78% and 48%, respectively, as discussed in Fig. S16. Table 1 lists the luminescence performances of some relevant Fe^3+^-doped and Cr^3+^/Yb^3+^ codoped phosphors. Although the measurement of IQE and EQE of ASBO:Fe^3+^ cannot work due to their weak emissions, with the energy extraction effects of Yb^3+^ codoping and strong CT absorption of Fe^3+^, the IQE ~ 78% and EQE ~ 48% of SSBO:0.02Fe^3+^,0.15Yb^3+^ are quite comparable to that of the most efficient Fe^3+^ NIR phosphors^27,30,36^ and that of recently reported Fe^3+^/Yb^3+^ and Cr^3+^/Yb^3+^ NIR-emitting phosphors^55–57^. These findings do suggest that the optimized ASBO:Fe^3+^,Yb^3+^ NIR-emitting phosphors hold significant potential for practical applications.

**Table 1:** Excitation and emission peak wavelengths, and IQE and EQE values of some Fe^3+^-doped, Fe^3+^/Yb^3+^ codoped and Cr^3+^/Yb^3+^ codoped NIR-emitting phosphor samples

Host	Type	λ_ex_ (nm)	λ_em_ (nm)	IQE (%)	EQE (%)	Ref.
Ca_2_InSbO_6_	Fe^3+^	340	935	87	68	^27^
Ca_2.5_Hf_2.5_Ga_2.5_Al_0.5_O_12_	Fe^3+^	410	770	38.6	―	^28^
ZnGa_2_O_4_	Fe^3+^	344	720	34.2	―	^29^
Sr_2_ScSbO_6_	Fe^3+^	334	894	63.1	54.2	^30^
Sr_9_Ga(PO_4_)_7_	Fe^3+^	330	915	6.6	5.3	^31^
CaLaLiTeO_6_	Fe^3+^	330	966	54.05	45	^36^
Li_2_ZnSiO_4_	Fe^3+^	300	750	62.7	―	^61^
NaScSi_2_O_6_	Fe^3+^	300	900	13.3	―	^62^
Ca_2_LuSbO_6_	Fe^3+^	336	927	69	―	^63^
SSBO	Fe^3+^	355	975	―	―	This work
BSBO	Fe^3+^	370	930	―	―	
LiInSbO_6_	Cr^3+^/Yb^3+^	492	998	10	4.76	^4^
Sr_9_Cr(PO_4_)_7_	Cr^3+^/Yb^3+^	485	978	55.4	28.4	^33^
Lu_0.2_Sc_0.8_BO_3_	Cr^3+^/Yb^3+^	463	990	73.6	23.7	^51^
Ca_2_LuZr_2_Al_3_O_12_	Cr^3+^/Yb^3+^	460	1030	77.2	30.8	^55^
LiScGeO_6_	Cr^3+^/Yb^3+^	466	970	76.3	36.2	^56^
LiScP_2_O_7_	Cr^3+^/Yb^3+^	450	1001	74	39	^57^
Sr_9_Ga(PO_4_)_7_	Fe^3+^/Yb^3+^	330	978	11.8	9.4	^31^
SSBO	Fe^3+^/Yb^3+^	355	973	78	48	This work
BSBO	Fe^3+^/Yb^3+^	380	1006	―	―	

#### Luminescence thermal stability and optical thermometry of ASBO:Fe^3+^,Yb^3+^ phosphors

**Fig. 5:**
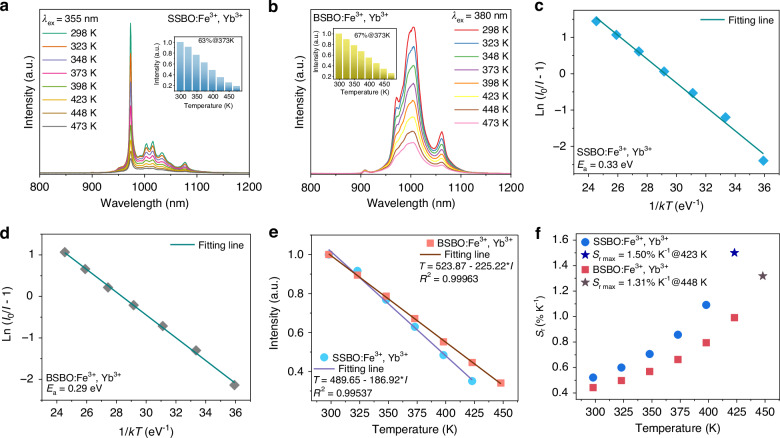
Luminescence thermal stability and thermometric properties of ASBO:Fe^3+^,Yb^3+^ (A = Sr, Ba). Temperature-dependent PL spectra of **a** SSBO:0.02Fe^3+^,0.15Yb^3+^ and **b** BSBO:0.02Fe^3+^,0.15Yb^3+^ phosphors. The insets show their corresponding integral PL intensities normalized to the initial intensity at room temperature (~ 298 K). Fitting curve of Ln (*I*_0_/*I*-1) and 1/*kT* coordinate relationship for **c** SSBO:0.02Fe^3+^,0.15Yb^3+^ and **d** BSBO:0.02Fe^3+^,0.15Yb^3+^, respectively. **e** Linear fitting of PL intensities versus temperature for SSBO:0.02Fe^3+^,0.15Yb^3+^ (solid square) and BSBO:0.02Fe^3+^,0.15Yb^3+^ (solid round) phosphors. **f** The relative sensitivity *S*_*r*_ of temperature sensing over 298-473 K for SSBO:0.02Fe^3+^,0.15Yb^3+^ (solid square) and BSBO:0.02Fe^3+^,0.15Yb^3+^ (solid round), respectively

To probe the luminescence thermal stabilities of optimized ASBO:0.02Fe^3+^,0.15Yb^3+^ (A = Sr, Ba) phosphors, Fig. 5a, b shows their emission spectra versus temperature, respectively. It can be seen that, as temperature increases from 298 to 498 K, the NIR emissions of ASBO:0.02Fe^3+^,0.15Yb^3+^ all gradually decrease due to thermal quenching effects. At 373 K (approximately at the operating temperature of pc-LED), the integral PL intensity remained at 63% and 67% of initial intensity at 298 K for SSBO:0.02Fe^3+^,0.15Yb^3+^ (inset of Fig. 5a) and BSBO:0.02Fe^3+^,0.15Yb^3+^ (inset of Fig. 5b), respectively. A significant improvement was realized in comparison with the ASBO:0.02Fe^3+^ case (around 32% at 373 K). According to the Arrhenius equation (Eq. 6), the *E*_*a*_ value of thermal quenching was calculated to be about 0.33 eV for ASBO:0.02Fe^3+^,0.15Yb^3+^ (Fig. 5c) and ~ 0.29 eV was for BSBO:0.02Fe^3+^,0.15Yb^3+^ (Fig. 5d), which are both better than the ASBO:0.02Fe^3+^ samples (Fig. 3c). In principle, nonradiative transitions probability of the exposed 3d-electron layer of Fe^3+^ will be effectively increased with the temperature rising for a poor luminescence thermal stability, while conversely the well-shielded 4 f electron orbitals of Yb^3+^ are not easily affected, inducing a remarked thermal stability for the ASBO:Fe^3+^,Yb^3+^ (A = Sr, Ba) samples.

Luminescence thermal stability is crucial to the ultimate application of pc-LED, while the thermal sensing performance of the studied phosphors can be additionally utilized for optical thermometry to monitor the temperature of working device^58^. Fig. 5e exhibits the temperature-dependent integral PL intensities (normalized to the intensity at 298 K) of SSBO:0.02Fe^3+^,0.15Yb^3+^ and BSBO:0.02Fe^3+^,0.15Yb^3+^, respectively. It can be found that the variation can be well fitted by a linear equation as *T* = 489.65 – 186.92*I for SSBO:0.02Fe^3+^,0.15Yb^3+^ and as *T* = 523.87 – 225.22*I for BSBO:0.02Fe^3+^,0.15Yb^3+^, respectively. On basis of the derivation of optical thermometric characteristics, the value of relative sensitivity (*S*_r_) can be rationally evaluated as^59^

8 $${S}_{r}=\frac{1}{I}\frac{\Delta I}{\Delta T}\,\times 100 \%$$

where *I* and *T* represent the integral PL intensity and temperature, respectively. By calculation, the variation of *S*_r_ at different temperature was detailed in Fig. 5f for SSBO:0.02Fe^3+^,0.15Yb^3+^ and BSBO:0.02Fe^3+^,0.15Yb^3+^, respectively. The *S*_r_ value increases monotonically with rising temperature and reaches a maximum of 1.50% K^-1^ at 423 K (1.50% K^-1^@423 K) for SSBO:0.02Fe^3+^,0.15Yb^3+^ and of 1.31% K^-1^@448 K for BSBO:0.02Fe^3+^,0.15Yb^3+^, respectively. These results certify the potential application of ASBO:Fe^3+^,Yb^3+^ (A = Sr, Ba) phosphors in the field of optical thermometry, for instance, real-time monitoring the working temperature of pc-LED light source.

### Applications of NIR pc-LED light source

**Fig. 6:**
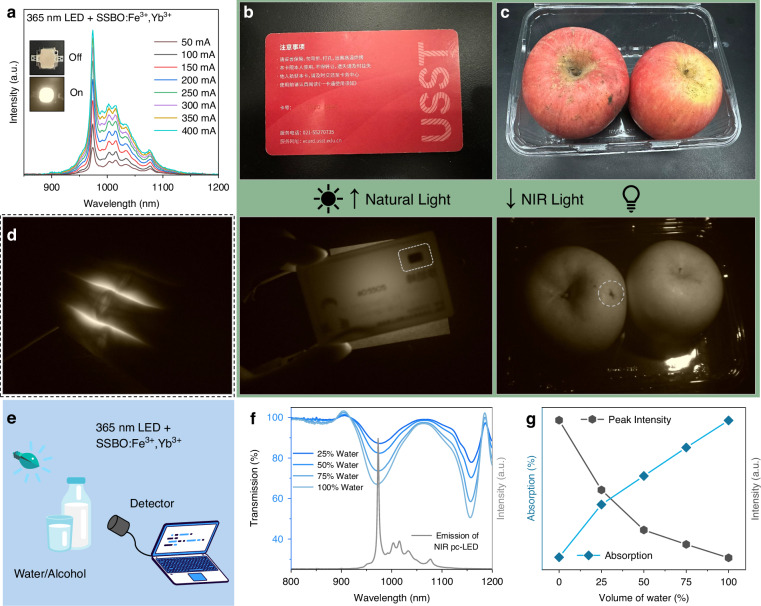
Multiple applications of SSBO:0.02Fe^3+^,0.15Yb^3+^-based NIR pc-LED. **a** Electroluminescence spectra of a home-made pc-LED by combining a 365 nm LED chip with SSBO:0.02Fe^3+^,0.15Yb^3+^ phosphor. The insets show photograph of the pc-LED with light off and that with light on taken by a NIR camera, respectively. **b** One IC card and **c** two apples (one having a surface flaw) photographed under natural light and under NIR light illuminated by the fabricated pc-LED, respectively. **d** Photograph of the NIR light penetrating human fingers. **e** Schematic diagram of liquid composition detection using NIR light sources. **f** Transmission spectra (blue curve) of alcohol solutions with different water volume fraction, and the NIR emission spectra of SSBO:0.02Fe^3+^,0.15Yb^3+^-based pc-LED. **g** Emission intensity of the home-made pc-LED light passing through alcohol solutions and solution absorption rate at peak wavelength ~ 973 nm versus water content volumes

A prototype pc-LED was fabricated by integrating the optimized SSBO:0.02Fe^3+^,0.15Yb^3+^ phosphors with a commercial ~365 nm UV chip, as illustrated in the inset of Fig. 6a. The resulting pc-LED produces a broadband electroluminescence (EL) spectrum spanning 850–1150 nm, which originates from the characteristic Yb^3+ 2^F_5/2_ → ^2^F_7/2_ transition. This NIR emission exhibits a marked enhancement in intensity as the driving current increases from 50 to 400 mA. The inset photograph, captured using a NIR camera equipped with an 850 nm long-pass filter, demonstrates the substantial short-wave infrared (SWIR) components available for practical applications. As shown in Fig. 6b, the NIR light from the pc-LED can nondestructively penetrate an IC card that is otherwise opaque to visible light, clearly unveiling the internal chip and the patterns on the back of the card. Figure 6c presents comparative images of two apples under natural and NIR illumination. Beyond simple identification, surface flaws are clearly discernible under NIR illumination, directly validating the device’s potential for night vision and food inspection. Furthermore, leveraging the effective penetration of NIR light through biological tissues, the system successfully resolved the subcutaneous vasculature within human fingers (Fig. 6d), providing robust support for its application in biomedical imaging.

To fully evaluate the utility of the fabricated pc-LED NIR light source, its performance in the field of NIR spectroscopic analysis was investigated. It is well-established that different organic functional groups exhibit distinct characteristic absorption bands within the NIR region. Consequently, colorless transparent liquids, such as ethanol and water, as well as their volume fractions, can be identified via spectroscopic analysis utilizing the prepared broadband pc-LED. The protocol for the NIR spectroscopic analysis of water-ethanol mixtures is schematically illustrated in Fig. 6e. During the experiment, the absorption spectra of ethanol solutions with varying water volume fractions were recorded (Fig. 6f). As the moisture content increases from 25% to 100%, a prominent absorption band emerges between 920 and 1070 nm (peaking at ~970 nm), which corresponds to the characteristic overtones of the O–H stretching vibrations in H_2_O. This band well aligns with the emission light of the fabricated pc-LED (represented by the green curve in Fig. 6f). Additionally, the absorption feature within the 900–920 nm range is attributed to the C–H stretching vibrations of ethanol^60^. Figure S15a displays the measured transmission spectra after the pc-LED NIR light passes through ethanol with varying water volumes; a clear decrease in the integrated intensity is observed (inset of Fig. S17a). This elevated water content exerts an observable inhibitory effect on the peak intensity of the transmitted NIR light (detailed in Fig. S17b). Finally, Fig. 6g illustrates the correlation between the solution absorption rate and the variation in PL intensity at the ~973 nm peak under different water concentrations. The results show that the fluctuations in absorption intensity induced by the water volume are highly consistent with the trend of emission intensity attenuation after NIR penetration. These findings demonstrate that the water content in ethanol solutions can be quantitatively estimated via changes in the NIR peak intensity, verifying the significant potential of phosphor-based pc-LEDs in spectroscopic analysis for component identification and concentration determination.

## Discussion

In summary, a series of mixed orthoborate-pyroborate phosphors ASBO:Fe^3+^,Yb^3+^ (A = Sr, Ba) were successfully prepared by means of a traditional high-temperature solid-state reaction at 1323 K. High crystallinity pure-phase ASBO:Fe^3+^,Yb^3+^ products were obtained with the identification of XRD measurements, Rietveld refinements, and SEM characterizations. Due to the O^2-^ → Fe^3+^ CT transition, the ASBO:Fe^3+^ samples have broadband absorption within 240-450 nm peaked at 355 and 370 nm for the SSBO and BSBO systems, respectively. Under UV excitation, the SSBO:Fe^3+^ and BSBO:Fe^3+^ samples efficiently yielded broadband emission at 975 and 930 nm with large FWHM values approximately 167 and 170 nm, readily attributed to the ^4^T_1_ (^4^G) → ^6^A_1_ (^6^S) transition of Fe^3+^ interacted with the electron phonon coupling effects. These new phosphors SSBO:Fe^3+^ and BSBO:Fe^3+^ have much longer PL wavelengths partially covering a desirable NIR-II window and wider FWHMs than most of recently reported Fe^3+^ NIR phosphors. On the other hand, the ASBO:Fe^3+^,Yb^3+^ phosphors had about 160-fold enhancement of the integral PL intensity around 1000 nm compared to the ASBO:Fe^3+^ basis. The optimal SSBO:0.02Fe^3+^,0.15Yb^3+^ sample had comparable IQE ~ 78% and EQE ~ 48% even to the recently popular Cr^3+^/Yb^3+^-codoped NIR phosphors. Besides the resonant ET of Fe^3+^ → Yb^3+^, effective energy extraction of Yb^3+^ from the major dark Fe^3+^ ions were rationally proposed for the massively enhanced luminescence on basis of crystal structure of ASBO host, steady fluorescence spectra and lifetime data. Meanwhile, the ASBO:Fe^3+^,Yb^3+^ phosphors were determined to have much superior PL stability, > 63%@373 K, to the ASBO:Fe^3+^ phosphors (~ 32%@373 K). For temperature sensing, the SSBO:0.02Fe^3+^,0.15Yb^3+^ and BSBO:0.02Fe^3+^,0.15Yb^3+^ exhibited applicable *S*_*r*_ about 1.5% K^-1^@423 K and 1.31% K^-1^@448 K, respectively. Ultimately, an SSBO:0.02Fe^3+^,0.15Yb^3+^-based pc-LED NIR light source was successfully fabricated and used to clearly display the internal chip and patterns of an IC card, to image apple (matter) in the dark and pick out the surface flaws, to recognize blood vessel distribution inside human fingers, and to achieve NIR spectroscopy analysis. The current results offer profound insights into the design and fabrication of new generation of Fe^3+^-based NIR-emitting phosphors and the relevant pc-LED NIR light source.

## Materials and methods

### Synthesis

A series of A_2_Sc_2-x-y_B_4_O_11_:*x*Fe^3+^,*y*Yb^3+^ (A = Sr or Ba, *x* = 0-0.1, *y* = 0-0.3) (A = Sr or Ba, *x* = 0-0.1, *y* = 0-0.3) polycrystalline phosphors were synthesized through a simple high-temperature solid-state reaction in air atmosphere. Starting materials of SrCO_3_ (99.99%, Aladdin), BaCO_3_ (99.99%, Aladdin), Sc_2_O_3_ (99.99%, Aladdin), H_3_BO_3_ (99.9%, Aladdin), Fe_2_O_3_ (99.99%, Aladdin) and Yb_2_O_3_ (99.99%, Aladdin) were used without further purification. All chemicals were carefully weighed according to the nominally stoichiometric compositions, and then thoroughly mixed and ground in an agate mortar for about 20 min. Finally, the resultant powder mixtures were completely transferred into aluminum crucibles, respectively, which were placed in a muffle furnace for sintering at 1323 K with a temperature ramping rate of 5 K min^-1^. Noted that, for the batch of Sr_2_Sc_2_B_4_O_11_:*x*Fe^3+^,*y*Yb^3+^ polycrystalline phosphors, the sintering at 1323 K was kept for 12 h, while for the batch of Ba_2_Sc_2_B_4_O_11_:*x*Fe^3+^,*y*Yb^3+^ phosphors, the sintering process was practically sustained for 6 h. After naturally cool down to room temperature, all the prepared products were crushed and ground into fine powders for versatile characterizations and spectral testing.

### Fabrication of NIR pc-LED prototype

The NIR pc-LED device was fabricated with an Epoxy Resin obtained from Shenzhen Juhengchuang Electronic Materials Co., Ltd., and using a 10 W UV LED chip from Shenzhen Fangpu Optoelectronics Co., Ltd. Specifically, the optimized Sr_2_Sc_2_B_4_O_11_:0.02Fe^3+^,0.15Yb^3+^ phosphor was thoroughly mixed with resins A and B with ratio of A:B = 1:1. Then the mixture was coated on the 365 nm LED chip, and cured at 100 °C for 3 h to fabricate the final NIR pc-LED light source for applications. The operating current source is a direct-current power supply (UTP 1360S).

### Characterization

Phase identification of all as-prepared ASBO:*x*Fe^3+^,*y*Yb^3+^ (A = Sr or Ba) phosphor samples were performed using a Rigaku MiniFlex600 powder X-ray diffractometer (Cu Kα radiation, λ = 1.5406 Å) at 40 kV and 15 mA to collect the X-ray diffraction (XRD) patterns. For Rietveld refinements to acquire detailed information on the phase compositions and cell parameters, the XRD patterns were additionally determined by a Bruker D2 Phaser (AXS, Germany) with a slow scanning speed of 0.02° in the 10–80° range using Cu Kα radiation (λ = 1.5418 Å) at 30 kV and 10 mA. To characterize the size distribution, morphology and elemental mapping of SSBO:0.02Fe^3+^,0.15Yb^3+^ representative sample, a trace amount of powder sample was taken and sprinkled to be a very thin layer onto the conductive adhesive (carbon tape) on an aluminum stub. After removing excess powders from the adhesive surface using a gentle stream of clean and dry air, a 5 nm thick gold film was sprayed at a current of 30 mA in the chamber of a vacuum coating instrument. Then, the microscopic morphology and elemental mapping were measured by a ZEISS scanning electron microscope (SEM, JSM-IT500HR, Japan) with energy-dispersive X-ray spectrometry (EDS) at 15 KV and 41 *μ*A. Moreover, X-ray photoelectron spectroscopy (XPS) spectra were collected on a Thermo Scientific K-alpha X-ray photoelectron spectrometer. The Fe K-edge XANES measurement was conducted at the hard X-ray beamline (BL13U) of the Shanghai Synchrotron Radiation Facility (SSRF). The photon energy was scanned across the Fe K absorption edge from 7.08 to 7.22 keV using a double-crystal monochromator with a step size of 0.2 eV and an estimated bandwidth of ~0.7 eV. The SSBO-based sample and a Fe₂O₃ standard reference sample were measured and an XANES data of FeO was from the IXAS X-ray Absorption Data Library measured by the beamline 20-BM-B of the Advanced Photon Source (APS).

By employing a Shimadzu SolidSpec-3700 DUV-UV-Visible-NIR spectrophotometer, diffuse reflectance spectra of as-obtained ASBO:Fe^3+^,Yb^3+^ phosphors were measured in 200-1200 nm with the BaSO_4_ powder as the reference standard. Steady-state PL and PLE spectra of all the samples were recorded on an FLS1000 fluorescence spectrophotometer (Edinburgh Instruments, UK) with a 450 W xenon lamp and a liquid-nitrogen-cooled NIR R5509-72 photomultiplier tube (Hamamatsu Corp.). Luminescence decay curves and the temperature-dependent emission spectra were measured on the FLS1000 system with help of a *μ*F900 microsecond pulsed xenon lamp and a precision temperature-controlled instrument (INSTEC HP1200 G, US), respectively. Measurements of IQE, EQE, and absorption coefficient for the SSBO:Fe^3+^,Yb^3+^ NIR-emitting phosphors were carried out on an absolute PL quantum yield measurement system (Quantaurus-QY Plus C13534-11 with a unit C13684-01, Hamamatsu Photonics). Photographs were taken under NIR light and natural light using a Vis-NIR camera with and without an 850 nm long pass filter, respectively. The camera is manufactured by Panasonic and carries the model number DMC-GF3.

## Supplementary information

Supporting Information

## Data Availability

All the data supporting this study are presented in the “Results” section and Supplementary Information and are available from the corresponding authors upon reasonable request.
