# Supplementary material for: Energy extraction from dark Fe3+ in A2Sc2B4O11:Fe3+, Yb3+ (A = Sr, Ba) toward promoted NIR luminescence and pc-LED light source for multifunctional applications
Source: Light Sci Appl. 2026 May 9;15:229. doi: 10.1038/s41377-026-02284-8 (PMC13157481; doi:10.1038/s41377-026-02284-8)
Supplement: Supplementary file 1 — Supporting Information [file 41377_2026_2284_MOESM1_ESM.docx]

**Supporting Information**

**Energy extraction from dark Fe^3+^ in A_2_Sc_2_B_4_O_11_:Fe^3+^,Yb^3+^ (A = Sr, Ba) toward promoted NIR luminescence and pc-LED light source for multifunctional applications**

Dechao Yu,^1,*^ Haisheng Liu,^1^ Mengting Lv,^1^ Benchun Li,^1^ Yayun Zhou,^2^ Xinxin Han,^3^ and Dawei Zhang^1,*^

*^1^Engineering Research Center of Optical Instrument and System, Ministry of Education and Shanghai Key Lab of Modern Optical System, University of Shanghai for Science and Technology, Shanghai 200093, PR China*

*^2^Guangdong-Hong Kong-Macao Joint Laboratory for Intelligent Micro-Nano Optoelectronic Technology, School of Physics and Optoelectronic Engineering, Foshan University, Foshan 528225, PR China*

*^3^MOE Key Laboratory of New Processing Technology for Non-ferrous Metals and Materials, Guangxi Key Laboratory of Processing for Non-ferrous Metals and Featured Materials, Guangxi University, Nanning, 530004, China*

*Corresponding authors: Dechao Yu, [d.yu@usst.edu.cn](mailto:d.yu@usst.edu.cn); Dawei Zhang, [dwzhang@usst.edu.cn](mailto:dwzhang@usst.edu.cn)


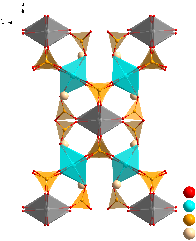

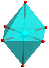

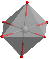


Sc1O_6_

Sc2O_6_

O

Sc

B

Ba


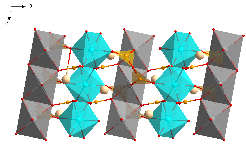


3.17 Å


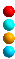


O

Sc

B

Sr


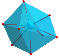

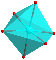


ScO_6_

SrO_8_


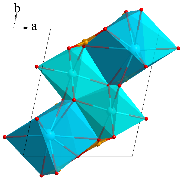


3.26 Å

a

b

**Fig. S1** Crystal structure and coordination diagram of **a** SSBO host and **b** BSBO host.

a

b

c

**Fig. S2** XRD patterns of **a** SSBO:*x*Fe^3+^ (*x* = 0.001-0.05), **b** BSBO:*x*Fe^3+^ (*x* = 0.01-0.05) and **c** SSBO:0.02Fe^3+^, *y*Yb^3+^ (*y* = 0.01-0.3), as well as that of SSBO (PDF#89-1563) and BSBO (PDF#89-1564) standard reference.

a

b

c

**Fig. S3** XRD Rietveld refinement of as-prepared **a** SSBO:0.02Fe^3+^, **b** BSBO:0.02Fe^3+^ and **c** BSBO:0.02Fe^3+^,0.15Yb^3+^ phosphor samples, respectively.


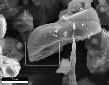

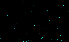

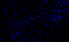

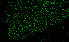

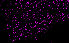

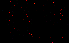


**B**

**O**

**Sr**

**Sc**

**Fe**

**5 μm**

a


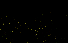

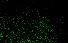

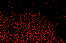

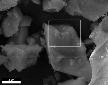

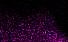


**O**

**Ba**

**Sc**

**Fe**

**2 μm**


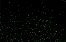


**B**

b


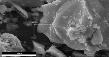


**O**

**Ba**

**Sc**

**Fe**

**5 μm**

**B**

c


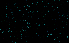


**B**


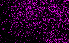


**O**


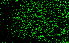


**Sc**


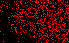


**Ba**


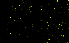


**Fe**


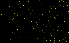


**Yb**

**Fig. S4** SEM and elemental mapping images of as-obtained **a** SSBO:0.02Fe^3+^, **b** BSBO:0.02Fe^3+^ and **c** BSBO:0.02Fe^3+^,0.15Yb^3+^ phosphor samples, respectively.

c

a

d

b

**Fig. S5** **a** Comparison of emission spectrum of SSBO:0.02Fe^3+^ (red area) with that of BSBO:0.02Fe^3+^ (blue area). **b** Emission spectra of SSBO:*x*Fe^3+^ (*x* = 0.001-0.05), and **c** the corresponding integral emission intensity (red dotted curve) and FWHM values (histogram) as a function of Fe^3+^-doping concentration. **d** Normalized excitation spectra of SSBO:*x*Fe^3+^ (*x* = 0.001-0.01).

a

b

**Fig. S6 a** Emission spectra of and **b** Normalized excitation spectra of BSBO:*x*Fe^3+^ (*x* = 0.01-0.05).

**Fig. S7** Calculated optical bandgap values of SSBO host and BSBO host.

The optical bandgap can be calculated using the Tauc and Kubelka Munk equations as follows^1,2^:

$$\begin{aligned} F\left( R \right)=\frac{\left( 1-R \right)^{2}}{2R}\#\left( S1 \right) \end{aligned}$$

$$\begin{aligned} {[F(R)hv]}^{1/n}=A\left( hv-E_{g} \right)\#\left( S2 \right) \end{aligned}$$

where *F(R), R*, *hv*, *A*, and *E*_g_ represent absorption coefficient, reflectance, photon energy, constant, and bandgap, respectively. For the host which is direct bandgap semiconductor, the value of n is 1/2, while for the host which is indirect bandgap semiconductor, the value of n is 2. Here, SSBO host and BSBO host are indirect bandgap semiconductors (n = 2). The bandgap *E*_g_ can be determined by the intersection of the tangent line of the curve in the figure and the photon energy *hv* axis.

a

b

**Fig. S8** Luminescence decay curves of **a** SSBO:0.02Fe^3+^ and **b** BSBO:0.02Fe^3+^ monitoring at different emission wavelengths under excitation of 355 and 370 nm, respectively.

a

b

**Fig. S9** **a** Gaussian peak fitting results of emission spectra centered 930 nm (~10752 cm^-1^) and **b** excitation spectra monitoring at different emission wavelengths of BSBO:0.02Fe^3+^.

a

b

c

**Fig. S10** Emission spectra of **a** SSBO:0.02Fe^3+^ and **b** BSBO:0.02Fe^3+^ at low-temperature (80 K) and room temperature (298K). **c** Gaussian peak fitting results of emission spectra centered 948 nm (~10548 cm^-1^) of BSBO:0.02Fe^3+^ at 80 K.

**Fig. S11** Normalized excitation spectra of BSBO:0.02Fe^3+^ and BSBO:0.02Fe^3+^,0.15Yb^3+^, respectively.

a

b

c

**Fig. S12** **a** Excitation spectra of SSBO:0.02Fe^3+^,0.15Yb^3+^ and SSBO:0.15Yb^3+^ monitoring emission wavelength at 973 nm, as well as their normalization. **b** NIR emission spectra of SSBO:0.02Fe^3+^,0.15Yb^3+^ and SSBO:0.15Yb^3+^ under excitation of 355 nm, respectively. **c** Excitation spectra and NIR emission spectra of BSBO:0.02Fe^3+^,0.15Yb^3+^ and BSBO:0.15Yb^3+^.

**Fig. S13** Comparison of emission spectrum of SSBO:0.02Fe^3+^,0.15Yb^3+^ (red area) and BSBO:0.02Fe^3+^,0.15Yb^3+^ (blue area) phosphors. For rigorous comparison, emission spectra of the same batch of samples were measured under the same excitation conditions except wavelengths.

The observed significant discrepancy in the emission shapes between SSBO:Fe^3+^,Yb^3+^ and BSBO:Fe^3+^,Yb^3+^ (as shown in Fig. S13) can be attributed to the specific crystallographic environments of the Yb^3+^ ions. The distinct spectral features of Yb^3+^ at different sites are governed by site symmetry and selection rules. The low-symmetry 2i Sc^3+^ site (in SSBO) lacks an inversion center, allowing sharp electric dipole transitions to dominate the spectrum. In contrast, the high-symmetry 4a/b site (in BSBO) possesses an inversion center ($1$), which strictly forbids pure electric dipole transitions. Consequently, the emission from this site is characterized by broad vibronic side bands (phonon-assisted transitions) and magnetic dipole lines. This interpretation aligns with the mechanism of vibronic coupling in centrosymmetric sites described by Blasse **^3^** and the comparative study of non-centrosymmetric (C_2_) versus centrosymmetric (C_3i_) sites in Y_2_O_3_ by Gruber et al. ^4^

**Fig. S14** Normalized excitation spectra of SSBO:0.02Fe^3+^,*y*Yb^3+^ (*y* = 0.01-0.3) monitoring emission at 973 nm.

**Fig. S15** Luminescence decay curves of SSBO:0.02Fe^3+^,*y*Yb^3+^ (*y* = 0.01-0.3) phosphor samples.

**Fig. S16** Excitation line of BaSO_4_ and emission spectrum of the SSBO:0.02Fe^3+^,0.15Yb^3+^ phosphor sample collected using an integrating sphere. The inset shows a magnification of the emission spectrum.

Internal quantum efficiency (*η*_i_), absorption efficiency (*η*_a_) and external quantum efficiency (*η*_e_) are calculated using the following equations, respectively:

$$\begin{aligned} \eta_{i}=\frac{\int L_{s}}{\int E_{R}-\int E_{S}}\#\left( S3 \right) \end{aligned}$$

$$\begin{aligned} \eta_{abs}=\frac{\int E_{R}-\int E_{S}}{\int E_{R}}\#\left( S4 \right) \end{aligned}$$

$$\begin{aligned} \eta_{e}=\eta_{i}*\eta_{abs}\#\left( S5 \right) \end{aligned}$$

where *E*_R_ is the spectrum of the excitation light without the sample in the sphere, *E*_S_ is the spectrum of the light used for exciting the sample, and *L*_S_ is the emission spectrum of the studied sample. From the information provided, it follows that the value of internal quantum efficiency is 78%, the values of absorption efficiency is 61% and the values of external quantum efficiency is 48% for SSBO:0.02Fe^3+^,0.15Yb^3+^ phosphors.

a

b

**Fig. S17** **a** Emission spectra of NIR light generated by the constructed pc-LED passing through alcohol solutions with different water volume fractions. Inset plots the integral intensities over the whole NIR PL band as a function of water volume fraction. **b** Emission spectra of the NIR light emitted by the constructed pc-LED passing through ethanol solutions with different water volume fractions.

**Table S1** Refined structural parameters and cell parameter values of ASBO:Fe^3+^,Yb^3+^ (A = Sr, Ba).

| Phosphor | Space Group | Parameters (Å) | Volume (Å^3^) | *R* |
| --- | --- | --- | --- | --- |
| SSBO standard | P$\bar{1}$ | *a* = 6.293  *b* =7.285  *c* = 5.084 | 220.4 | ― |
| SSBO:0.02Fe^3+^ | P$\bar{1}$ | *a* = 6.2923(5)  *b* =7.28132(30)  *c* = 5.0815(3) | 220.260(9) | *R*_wp_ =10.275%  *R*_p_ = 7.787%  *χ*^2^ = 2.39 |
| SSBO:0.02Fe^3+^,0.15Yb^3+^ | P$\bar{1}$ | *a* = 6.3020(9)  *b* = 7.2948(5)  *c* = 5.0867(6) | 221.354(18) | *R*_wp_ = 10.555%  *R*_p_ = 8.02%  *χ*^2^ = 2.89 |
| BSBO standard | C2/c | *a* = 16.022  *b* = 9.354  *c* = 6.342 | 937.8 | ― |
| BSBO:0.02Fe^3+^ | C2/c | *a* = 16.0178(13)  *b* = 9.3549(3)  *c* = 6.36039(31) | 937.26(8) | *R*_wp_ = 13.539%  *R*_p_ = 10.44%  *χ*^2^ = 2.65 |
| BSBO:0.02Fe^3+^,0.15Yb^3+^ | C2/c | *a* =16.0291(39)  *b* = 9.3584(5)  *c* = 6.3624(72) | 938.43(2) | *R*_wp_ = 14.637%  *R*_p_ = 11.44%  *χ*^2^ = 2.64 |

**Table S2** Excitation peak wavelength **(**λ_ex_**)**, emission peak (λ_em_) and FWHM values of some Fe^3+^-activated NIR-emitting phosphors.

| Phosphor | λ_ex_ (nm) | λ_em_ (nm) | FWHM (nm) | *I_T_*/*I*_298K_ | *E*_a_ (eV) | Ref. |
| --- | --- | --- | --- | --- | --- | --- |
| Sr_2_ScSbO_6_:Fe^3+^ | 334 | 894 | 73 | 81% (423K) | ― | 5 |
| Ca_2.5_Hf_2.5_Ga_2.5_Al_0.5_O_12_:Fe^3+^ | 410 | 770 | 100 | ~30% (373K) | ― | 6 |
| Ca_2_InSbO_6_:Fe^3+^ | 340 | 935 | 126 | 36% (398K) | 0.31 | 7 |
| CaLaLiTeO_6_:Fe^3+^ | 330 | 966 | 146 | 49% (373K) | 0.40 | 8 |
| Ca_2_LuSbO_6_:Fe^3+^ | 336 | 927 | 128 | 30% (393K) | ― | 9 |
| NaScSi_2_O_6_:Fe^3+^ | 300 | 900 | 135 | 23% (433K) | ― | 10 |
| Li_2_ZnSiO_4_:Fe^3+^ | 300 | 750 | 78 | 35% (373K) | 0.47 | 11 |
| Sr_9_Ga(PO_4_)_7_:Fe^3+^ | 330 | 915 | 155 | ＜30% (373K) | 0.18 | 12 |
| ZnGa_2_O_4_:Fe^3+^ | 344 | 720 | 70 | 71% (423K) | 0.37 | 13 |
| CaAl_12_O_19_:Fe^3+^ | 280 | 808 | 50 | ― | ― | 14 |
| NaAl_5_O_8_:Fe^3+^ | 346 | 754 | 75 | 83% (423K) | 0.23 | 15 |
| CaAl_4_O_7_:Fe^3+^ | 350 | 767 | 100 | ― | ― | 16 |
| BSBO:Fe^3+^ | 355 | 930 | 170 | 32% (373K) | 0.30 | This work |
| SSBO:Fe^3+^ | 370 | 975 | 166 | 32% (373K) | 0.27 |  |

**Table S3** Fitting parameters (*A*_1_, *A*_2_), component lifetimes (*τ*_1_, *τ*_2_), the calculated decay times (*τ*) and R-squared (*R*^2^) of decay curves as a bi-exponential function of different emission wavelengths in BSBO:0.02Fe^3+^ under excitation at 370 nm.

| λ_em_ (nm) | *A*_1_ | *τ*_1_ (*μ*s) | *A*_2_ | *τ*_2_ (*μ*s) | *τ* (*μ*s) | *R*^2^ |
| --- | --- | --- | --- | --- | --- | --- |
| 850 | 1.0573 | 24.0132 | 0.0833 | 221.8106 | 107.3200 | 0.9934 |
| 900 | 1.0632 | 25.3943 | 0.0626 | 212.5438 | 87.1497 | 0.9977 |
| 930 | 0.9039 | 25.4391 | 0.0534 | 176.5969 | 69.3838 | 0.9983 |
| 941 | 0.8887 | 24.9602 | 0.0478 | 170.9476 | 64.2601 | 0.9982 |
| 1009 | 1.0754 | 24.9850 | 0.0420 | 167.6473 | 54.6201 | 0.9978 |
| 1050 | 1.0321 | 22.9400 | 0.0634 | 99.7789 | 39.1393 | 0.9972 |
| 1100 | 0.8011 | 17.0685 | 0.3714 | 39.2972 | 28.5461 | 0.9960 |

The average lifetime (*τ*) for the bi-exponential decay curves was calculated using the following equation:

$$\begin{aligned} \tau=\frac{A_{1}\tau_{1}^{2}+A_{2}\tau_{2}^{2}}{A_{1}\tau_{1}+A_{2}\tau_{2}} \#\left( S6 \right) \end{aligned}$$

where *τ*_1_ and *τ*_2_ represent the component lifetimes, and *A*_1_ and *A*_2_ are the corresponding weighting factors.

**References:**

1. Yan, Y. et al. Photoluminescence properties of AScSi_2_O_6_: Cr^3+^ (A= Na and Li) phosphors with high efficiency and thermal stability for near-infrared phosphor-converted light-emitting diode light sources. *ACS Appl. Mater. Interfaces* **14**, 8179-8190 (2022).

2. Zhang, H. S. et al. Efficient and thermally stable broad-band near-infrared emission in a KAlP_2_O_7_: Cr^3+^ phosphor for nondestructive examination. *ACS Appl. Mater. Interfaces* **14**, 11663-11671 (2022).

3. Blasse, G. Vibronic transitions in rare earth spectroscopy. *Int. Rev. Phys. Chem.* **11**, 71–100 (1992).

4. Gruber, J. B., Leavitt, R. P., Morrison, C. A. & Chang, N. C. Optical spectra, energy levels, and crystal-field analysis of tripositive rare earth ions in Y2O3. IV. C_3i_ sites. *J. Chem. Phys.* **82**, 5373 (1985).

5. Zhang, X. B. et al. Tailoring Fe^3+^‐activated broadband NIR phosphors: enhancing external quantum efficiency and spectrum adjustability through crystal field engineering in double perovskite antimonate structures. *Adv. Opt. Mater.* **12**, 2302300 (2024).

6. Yan, L. Q. et al. Emerging Fe^3+^ doped broad NIR‐emitting phosphor Ca_2.5_Hf_2.5_(Ga, Al)_3_O_12_: Fe^3+^ for LWUV pumped NIR LED. *Laser Photonics Rev.* **18**, 2301200 (2024).

7. Liu, D. J. et al. Highly efficient Fe^3+^-doped A_2_BB′O_6_ (A= Sr^2+^, Ca^2+^; B, B′= In^3+^, Sb^5+^, Sn^4+^) broadband near-infrared-emitting phosphors for spectroscopic analysis. *Light Sci. Appl.* **11**, 112 (2022).

8. Su, S. K. et al. Achieving broadband NIR emission in Fe^3+^‐activated ALaBB′O_6_ (A= Ba, Sr, Ca; B–B′= Li–Te, Mg–Sb) phosphors via multi‐site ionic co‐substitutions. *Adv. Opt. Mater.* **12**, 2302383 (2024).

9. He, X. et al. Broadband emitting Fe^3+^-doped double perovskite-type antimonates phosphors for NIR spectroscopy applications. *J. Am. Ceram. Soc.* **107**, 2371-2383 (2024).

10. Zhang, X. G. et al. Broadband near-infrared luminescence from Fe^3+^-activated NaScSi_2_O_6_ phosphors for luminescence thermometry and night-vision applications. *Dalton Trans.* **51**, 14243-14249 (2022).

11. Yang, Y. et al. Tetracoordinate Fe^3+^ activated Li_2_ZnAO_4_ (A= Si, Ge) near-infrared luminescent phosphors. *Inorg. Chem.* **62**, 12862-12871 (2023).

12. Zhao, F. Y., Shao, Y. H., Song, Z. & Liu, Q. L. Structural confinement toward suppressing concentration and thermal quenching for improving near-infrared luminescence of Fe^3+^. *Inorg. Chem. Front.* **10**, 6701-6710 (2023).

13. Xiang, L. et al. Environmentally-friendly and low-cost Fe^3+^-doped broadband NIR light-emitting phosphors. *J. Lumin.* **252**, 119293 (2022).

14. Li, Y. J., Ma, Y. Y., Ye, S., Hu, G. P. & Zhang, Q. Y. Site-related near-infrared luminescence in MAl_12_O_19_ (M= Ca, Sr, Ba): Fe^3+^ phosphors. *Mater. Res. Bull.* **51**, 1-5 (2014).

15. Cheng, K., Huang, W. C., Liu, X. Y., Gong, X. Y. & Deng, C. Y. Cr^3+^-free broadband near-infrared phosphors NaAl_5_O_8_: Fe^3+^. *J. Alloys Compd.* **964**, 171240 (2023).

16. Kniec, K. et al. From quencher to potent activator–Multimodal luminescence thermometry with Fe^3+^ in the oxides MAl_4_O_7_ (M= Ca, Sr, Ba). *J. Mater. Chem. C* **9**, 6268-6276 (2021).
